# Mannan-binding lectin and procalcitonin measurement for prediction of postoperative infection

**DOI:** 10.1186/cc3768

**Published:** 2005-07-19

**Authors:** Michael Siassi, Jutta Riese, Rudi Steffensen, Michael Meisner, Steffen Thiel, Werner Hohenberger, Joachim Schmidt

**Affiliations:** 1 Department of Surgery, University Hospital Erlangen, Erlangen, Germany; 2 Regional Centre for Blood Transfusion and Clinical Immunology, Aalborg Hospital, Aalborg, Denmark; 3 Department of Anaesthesiology, University Hospital Jena, Jena, Germany; 4 Department of Medical Microbiology and Immunology, University of Aarhus, Aarhus, Denmark; 5 Professor, Department of Surgery, University Hospital Erlangen, Erlangen, Germany; 6 Department of Anaesthesiology, University Hospital Erlangen, Erlangen, Germany

## Abstract

**Introduction:**

Postoperative infection is a major cause of morbidity and mortality. We investigated two serum markers for their ability to identify patients at risk for postoperative infection. Mannan-binding lectin (MBL) is a central molecule of the innate immune system and MBL deficiency is known to predispose to infection. Procalcitonin (PCT) is a sensitive marker for bacterial infection.

**Methods:**

We investigated 162 patients undergoing elective surgery for cancer of the gastrointestinal tract. Patients were classified as having no complications (group A), having infection for unknown reason (group B) or having sepsis after events like aspiration or anastomotic leakage (group C). Analysis was done pre- and postoperatively for serum levels of MBL, PCT and C-reactive-protein. DNA was preoperatively sampled and stored and later analysed for genetic polymorphisms of MBL.

**Results:**

The preoperative serum levels of MBL were significantly lower in group B patients than in group A patients (1332 ± 466 ng/ml versus 2523 ± 181 ng/ml). PCT measured on day one post-surgery was significantly higher in group B patients than in group A (3.33 ± 1.08 ng/ml versus 1.38 ± 0.17 ng/ml). Patients with an aberrant MBL genotype had a significantly higher risk of postoperative infections than wild-type carriers (p < 0.05).

**Conclusion:**

Preoperative MBL and early postoperative PCT measurement may help identify patients at risk for postoperative infection.

## Introduction

Infection is a major reason for postoperative morbidity and mortality. Despite the use of new treatment modalities, improvements in technology and increased experience, morbidity rates are high and sepsis is the most common reason for mortality in surgical intensive care units [1]. Infection and sepsis in surgical patients occurs for various reasons. Some infections can be attributed to distinct events leading to an overwhelming bacterial load that would cause sepsis even in healthy persons, such as anastomotic leakage and aspiration (group C in this study). In other patients, however, an initial source of infection is not apparent, but it still occurs (group B in this study). Events of this type include bactaeremia of unknown reason, hospital acquired pneumonia, infection of in-dwelling catheters and bacterial translocation through the enteral mucosa. In contrast to the first group, these patients are exposed to a bacterial load that can normally be counteracted by the immune system even in the state of acute-phase metabolism. A compromised immune response of the host may predispose to clinically serious courses of infection. Various markers, including C-reactive protein (CRP), tumor necrosis factor α, IL-1, IL-6 and IL-8, have been studied for their ability to predict, diagnose and to differentiate infection, systemic inflammatory response syndrome and sepsis [2-4]. These markers have in common that they indicate activation of the immune system after infection has occurred. It is general surgical knowledge that postoperative infections usually occur after day 5 postoperatively, although there are no statistical data on this issue. Therefore, not only preoperative markers but also indicators in the early postoperative phase would be of predictive value.

Mannan-binding lectin (MBL) is a central part of the innate immune system. It belongs to a group of proteins called collectins. Its structure enables multiple binding to repeating oligosaccharide structures typical of bacterial surfaces [5]. After binding to micro-organisms, MBL activates the MBL-associated serine protease-2 and thus the lectin pathway of complement activation [6].

Previous studies have shown an increased susceptibility to bacterial, viral or fungal infections in patients with decreased MBL-serum levels [7,8]. A recent study identifed serum MBL level as an independent risk factor for survival in non-surgical intensive care unit patients [9]. The MBL concentration in serum is, in part, determined genetically. Some haplotypes confer low MBL concentrations or the secretion of non-functional protein. The main variants in exon 1 of the gene encoding MBL 2 are termed B, C and D variants, with A indicating the wild type. There are also polymorphisms in the 5' regulatory region at position -550 (H/L), -221 (X/Y variants) and in a 5' untranslated region at position +4 (P/Q variants). Due to linkage between polymorphisms, only seven common haplotypes exist with some leading to low MBL serum levels. Mutations in exon 1 (A/0 and 0/0 types) and the AX/AX type especially lead to low MBL serum levels. Altogether, depending on the disease studied, up to 25% of a Caucasian population may have insufficient MBL serum levels [10].

Procalcitonin (PCT), the prohormone of calcitonin, is normally produced in the C-cells of the thyroid gland and its concentration in the plasma of healthy subjects is very low (10–50 pg/ml) [11]. It is induced by bacterial endotoxin or inflammatory cytokines and both has a chemoattractant role and affects nitric oxide production.

PCT is preferentially induced during severe generalised bacterial, parasitic or fungal infections with systemic manifestations rather than in viral infections or inflammatory reactions of non-infectious origin [12].

In order to characterise patients with an increased susceptibility to postoperative sepsis, we studied the levels of MBL and PCT. To assess the influence of MBL polymorphisms, a genotype-analysis was performed. As a reference, a widely used marker of inflammation, CRP, was measured.

## Materials and methods

### Patients

We investigated patients undergoing major elective surgery for malignant disease of the gastrointestinal tract at the Department of Surgery in the University Hospital Erlangen (Erlangen, Germany) from January 1, 2000, until December 31, 2002. Demographic data for these patients is given in Table 1. Exclusion criteria were age below 18 years, pre-existing infection and emergency surgery. Patients were followed clinically until hospital discharge and postoperative complications were recorded according to the criteria of the American Council of Chest Physicians/Society of Critical Care Medicine [13]. Complications were termed 'postoperative infection' when signs of sepsis or systemic inflammatory response syndrome occurred with no obvious bacterial contamination or specific surgical problem (i.e. anastomotic leakage) (group B). Patients with other septic events were grouped separately (group C).

Blood samples were taken preoperatively on day 3 for MBL analysis and on day 1 and 3 postoperatively for PCT and CRP analysis; serum was stored at -76°C. The study was approved by the institutional ethics committee of the University of Erlangen.

### Measurement of MBL, PCT and CRP

MBL was measured by ELISA (Statens Serum Institut, Copenhagen, Denmark). MBL genotyping was performed using a real-time PCR assay on a LightCycler™ instrument (Roche Diagnostics, Mannheim, Germany). In this approach, PCR and melting temperature (*T*_m_) curve analysis are combined based on the principle of mutation detection by melting point analysis with a fluorescence resonance energy transfer hybridisation probe. The three mutations in exon 1 were detected in one capillary using a sensor probe covering the three mutations. Amplification of the variants located upstream of the coding sequence was performed by single colour detection for the H/L polymorphism and multiplexing by dual colour probes was used for simultaneous genotyping of X/Y and P/Q. The details of sample preparation and primer and probe design have been described elsewhere [14].

For statistical analysis, two groups were made. Group 1 included the genotypes leading to normal MBL levels; these are the homozygous wild-type carriers with the exception of the AX/AX type. Group 2 included all carriers of heterozygous or homozygous variations in exon 1 (A/O and O/O type) and the AX/AX type.

Serum PCT levels were determined by a specific and ultrasensitive immunoluminometric assay (Lumitest ProCa-S^®^, BRAHMS-Diagnostica, Berlin, Germany), which allowed measurement of the concentration of procalcitonin in human serum and plasma in the picogram range (5–5770 pg/ml) for diagnosis of locally restricted bacterial infections. Two monoclonal antibodies that bind PCT (the antigen) at two different binding sites (the calcitonin and katacalcin segments) were used. One of these antibodies (polyclonal, sheep) was luminescence labelled (the tracer), and the other (monoclonal, mouse) was fixed to the inner walls of the tube (coated tube system). During the course of incubation, both antibodies react with PCT molecules in the sample to form a sandwich. The luminescence signal is measured using a suitable luminometer and the LUMltest^® ^Basiskit reagents.

CRP-analysis was done by turbidimetry (Olympus, Hamburg, Germany).

### Statistical analysis

All serum levels are displayed as mean ± standard error of the mean (SEM). The statistical analysis was done using the t-test after logarithmic transformation of the raw data. CRP values were compared using the Mann-Whitney test. Correlation analysis was done using Spearman's rank correlation. All p-values are considered two-tailed. All tests were done using the SPSS 11.0 statistics software (SPSS, Munich, Germany).

## Results

Of the 172 patients included in the study, complete data for analysis were available for 162. Of these, 137 had no septic events (group A), 10 patients suffered from postoperative infections as defined above (group B) (characteristics are given in Table 2) and 15 patients had septic complications based on a defined postoperative event (group C) (Table 3).

The mean preoperative and postoperative MBL serum concentrations of all patients were 2462 ± 175 and 2375 ± 160 ng/ml, respectively (p = 0.6). The serum level of PCT rose from 0.24 ± 0.1 preoperative to 1.5 ± 0.17 ng/ml postoperative (p < 0.05).

**Figure 1:**
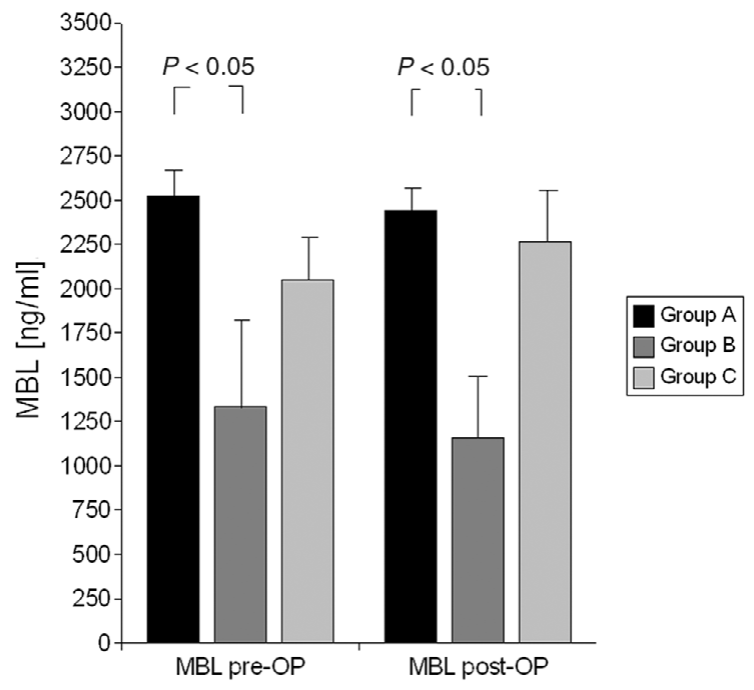
Comparison of pre- and postoperative serum-MBL in group A, B and C patients. Preoperative (pre-OP) and postoperative (post-OP) mannan-binding lectin (MBL) serum levels in patients with no postoperative infections (group A), with postoperative infections (group B) and with postoperative infections after a defined event (group C). Error bars indicate the standard error of the mean.

The mean preoperative MBL serum level in patients with postoperative infections (group B) was 1332 ± 466 ng/ml compared to 2523 ± 181 ng/ml in group A patients with no complications (p < 0.05). In patients who developed sepsis after a defined event (group C), preoperative MBL was 2047 ± 254 ng/ml, which did not differ significantly from group A. Postoperative MBL levels in group B and group A patients differed significantly at 1156 ± 393 ng/ml and 2442 ± 166 ng/ml, respectively (p < 0.05) (Fig. 1; complete data are given in Table 4).

**Figure 2:**
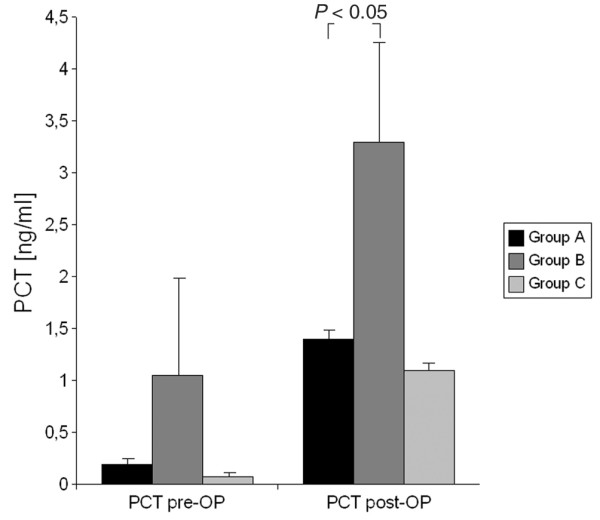
Comparison of pre- and postoperative serum-PCT in group A, B and C patients. Preoperative (pre-OP) and postoperative (post-OP) procalcitonin (PCT) serum levels in patients with no postoperative infections (group A), with postoperative infections (group B) and with postoperative infections after a defined event (group C). Error bars indicate the standard error of the mean.

The mean preoperative PCT level was 1.05 ± 1.0 ng/ml in patients with (group B) and 0.19 ± 0.1 ng/ml in patients without (group A) postoperative infection (p > 0.05). Postoperatively, there was a significant difference in PCT values between group B (3.33 ± 1.08 ng/ml) and group A (1.38 ± 0.17 ng/ml) (Fig. 2; complete data are given in Table 5).

Mean preoperative CRP was 16.0 ± 2.8 ng/l, 10.4 ± 3.0 ng/l and 11.1 ± 5.0 ng/ml in groups A, B and C, respectively (p > 0.05). On day 3 post surgery, the CRP values were 149.0 ± 5.9 ng/l, 209.4 ± 35.8 ng/l (p > 0.05) and 240.7 ± 22.3 ng/l (p < 0.05) in groups A, B and C, respectively. The measurement on day 1 did not show significant differences between the three groups.

**Figure 3:**
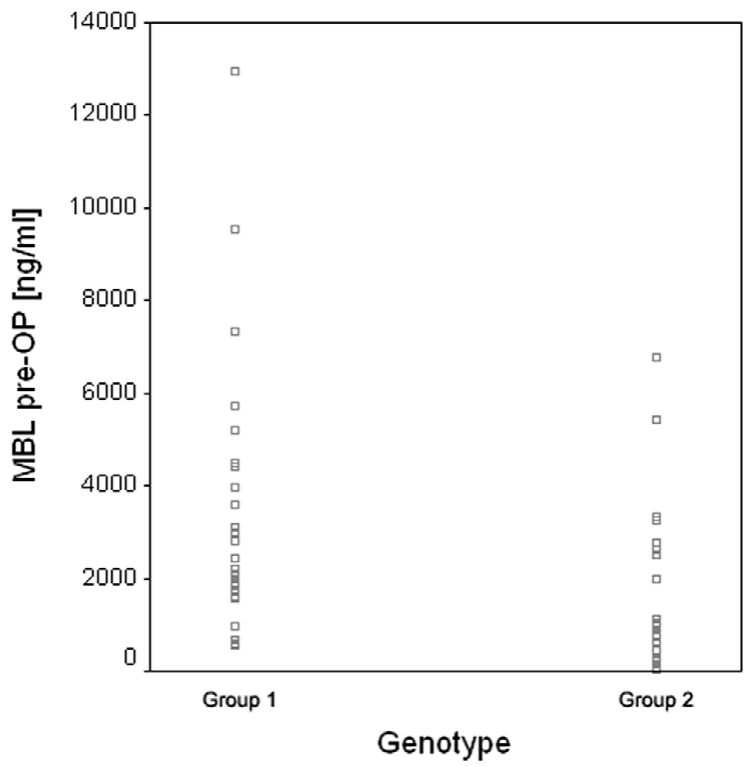
Correlation between MBL genotype group and MBL serum levels. Preoperative (pre-OP) mannan-binding lectin (MBL) levels in patients homozygous for the wild-type except the AX/AX type (group 1), and carriers of variations in exon 1 (A/O and O/O type) and the AX/AX type (group 2). Spearman rank correlation coefficient between genotype and preoperative serum MBL = -0.32, p = 0.02.

It was possible to perform a MBL genotype analysis in 59 patients. Patients carrying the A/A type but not the XA/XA type (group 1, n = 35) had a mean preoperative MBL level of 3097.1 ± 475.1 ng/ml, whereas the mean serum MBL in patients who were heterozygotic or homozygotic for any mutation in exon 1 (group 2; A/0 (n = 21) and XA/XA type (n = 3)) was 1794.0 ± 374.6 ng/ml (p = 0.04). The Spearman rank correlation coefficient between genotype group and preoperative serum MBL was -0.315 (p = 0.02) (Fig. 3). Of the group 1 and group 2 patients, 2/35 (6%) and 6/24 (25%) developed postoperative infections (group B), respectively (p = 0.035)

## Discussion

The search for a preoperative molecular marker defining patients at risk for postoperative infections is of great clinical interest because these patients may benefit from intensified monitoring. In this study, we show that low MBL serum levels and aberrant genotype are associated with a higher rate of postoperative infections. This correlates with earlier studies reporting a higher risk for infections in patients with MBL deficiency [7-9]. In contrast, a study in patients with fever of unknown cause showed no association between MBL deficiency and the course of infection [15]. In comparison to our study, however, the patient collective was not homogenous, with only fever as the primary entry criterion; the severity of sepsis differed substantially between patients, whereas the patient collective in our study was more homogenous. Also, this study only dealt with patients already having an infection and did not provide a 'control group' of patients not suffering from infection. The differences in the results between the two studies may, therefore, be due to different study designs and patient collectives. The risk of postoperative infection correlates with the type of surgery [16], which could cause bias. In our study, only patients undergoing elective surgery for gastrointestinal cancer were included. All patients underwent a resection of the gastrointestinal tract, causing some spillage of bacteria. The group was thus homogenous for the surgery-associated risk of infection.

An important issue in the design of our study was the distinction of patients who suffered complications leading to a bacterial challenge that would overwhelm even a normal immune system (group C) from patients with infection for unknown reason (group B). Mixing these cases in one group would lead to bias because immunological parameters may not play a great role in massive infection as it is encountered in group C patients.

In our study, serum MBL levels did not show a significant increase postoperatively. Postoperative MBL levels were also lower in patients with infections compared to those without. We could, therefore, not show an 'acute phase' like behaviour, as proposed in other studies. This may be due to the short postoperative phase investigated in our study. The previously described significant increase in postoperative MBL levels occurred on day 9 after surgery [17]. This late increase was not covered by our study design. Nevertheless, the comparison of procalcitonin and CRP as classic acute phase proteins and MBL showed a clear difference in their postoperative behaviour. We thus would not encourage the use of the term 'acute-phase protein' for MBL in the postoperative situation.

The analysis of the different genotypes of MBL showed a correlation between mutant genotypes and lower MBL serum levels as described before. The different genotypes were also strongly associated with postoperative infections; in our study, MBL serum levels and MBL genotyping showed similar correlation to infections in the samples that were tested for both. There are conflicting data on the clinical relevance of MBL mutations. A study on patients with pneumococcal disease showed an increased risk only in patients homozygous or functionally homozygous for MBL deficiency [7]. In contrast, a study on febrile neutropenia in children undergoing chemotherapy showed a clinical effect in patients with low MBL serum levels that was not limited to patients with exon 1 mutations [8]. Because the influence of serum levels on clinical outcome was the primary end-point in this study, we decided to group the serotypes according to their influence on MBL serum concentrations.

It has formerly been described that MBL genotyping is superior to the measurement of serum levels [18]. In our study, we show that, in the preoperative situation, measurement of MBL serum levels is as good a clinical marker as genotyping. Whereas the measurement of serum levels can easily be done by ELISA, MBL genotyping requires complex procedures that are not readily available in the clinical setting. This may facilitate future clinical use of MBL measurement.

Because MBL is now available both in a plasma-derived and a recombinant form, the question arises of whether supplementation in MBL-deficient individuals could minimise the risk of infections. The size of our study sample was too small to allow for multivariate analysis. We could not, therefore, identify MBL deficiency as an independent risk factor. The therapeutic use of high dose MBL in subjects with normal MBL levels must still be considered experimental and this approach should be addressed by larger studies.

In contrast to MBL, PCT showed no significant association between its preoperative serum level and the risk of postoperative infection. The trend towards higher preoperative PCT levels in group B patients may indicate pre-existing infection or systemic inflammatory response prior to surgery and needs further investigation.

Nevertheless, patients who developed infection had significantly higher PCT levels in the early postoperative phase. The measurement was made on day 1, whereas most infections occur later in the postoperative period. In contrast, CRP, which is widely used as a marker for infection, only showed a significant increase in group C patients on day 3. Because PCT is a sensitive marker of bacterial infection and systemic inflammation, this indicates that the actual bacterial load does not alone predispose to infection. It may instead show that the individual immune response plays a greater role. Despite the fact that preoperative PCT levels failed to predict infection, its early postoperative measurement (day 1) may help identify patients at risk for infection later on.

In our view, an immunologic factor that predisposes to infection can only play a role in an infection that occurs when the bacterial load is that of the average patient. In overwhelming infections caused by a massive bacterial load, those factors will not play a clinically significant role. Our results add new aspects to other studies that have shown increased susceptibility to infection in MBL-deficient individuals in non-surgical cases.

## Conclusion

Low preoperative MBL serum levels, as well as high PCT levels in the early postoperative phase, correlate with the occurence of postoperative infections. These markers may thus be useful for distinguishing patients at risk for infection. Prospective studies are needed to determine whether such patients benefit from intensified monitoring or prophylactic therapy.

## Key messages

○ Postoperative infection is a major cause for morbidity and mortality in gastrointestinal surgery.

○ Decreased serum MBL concentrations are associated with an increased risk of infection.

○ Preoperative MBL and early postoperative PCT measurement may help identify patients at risk for postoperative infections.

## Abbreviations

CRP = C-reactive protein; ELISA = enzyme-linked immunosorbent assay; IL = interleukin; MBL = mannan-binding lectin; PCR = polymerase chain reaction; PCT = procalcitonin; SEM = standard error of the mean.

## Competing interests

The authors declare that they have no competing interests.

## Authors' contributions

MS and JS conceived of the study, developed the study design, were responsible for patient recruitment and sample collection and carried out the statistical analysis. JR carried out the MBL serum measurements. RS performed the MBL genotyping. MM participated in the PCT analysis and ST participated in the design of the study and helped draft the manuscript. ST participated in the study design and data analysis. WH participated in the study design and drafting of the manuscript. All authors read and approved the final manuscript.

## Figures and Tables

**Table 1:** Patient demographic data

Age (years; mean and range)	62.3 (25–83)
Gender	
Female	56
Male	116
Type of surgery	
Resection of pancreas	28 (16.3%)
Gastrectomy	29 (16.9)
Esophageal resection	17 (9.9%)
Colorectal resection	98 (57.0%)

**Table 2:** Postoperative infections (group B)

Infection	Number of patients (n = 10)
Catheter sepsis	4
Pneumonia	2
Abdominal abscess	3
Urinary tract infection	1

**Table 3:** Other septic complications (group C)

Complication	Number of patients (n = 15)
Anatomotic leak	13
Aspiration	2

**Table 4:** Preoperative and postoperative serum levels of mannan-binding lectin

Group	Preoperative MBL (ng/ml)	Postoperative MBL (ng/ml)
All patients	2462 ± 175	2375 ± 160
Group A	2523 ± 181	2442 ± 166
Group B	1332 ± 466	1156 ± 393
Group C	2047 ± 254	2266 ± 389

MBL, mannan-binding lectin.

**Table 5:** Preoperative and postoperative serum levels of procalcitonin

Group	Preoperative PCT (ng/ml)	Postoperative PCT (ng/ml)
All patients	0.24 ± 0.1	1.5 ± 0.17
Group A	0.19 ± 0.1	1.4 ± 0.17
Group B	1.05 ± 1.0	3.3 ± 1.08
Group C	0.07 ± 0.04	1.1 ± 0.07

PCT, procalcitonin.
