# Correction: Mannan-binding lectin and procalcitonin measurement for prediction of postoperative infection

**DOI:** 10.1186/cc3820

**Published:** 2005-09-09

**Authors:** Michael Siassi, Jutta Riese, Rudi Steffensen, Michael Meisner, Steffen Thiel, Werner Hohenberger, Joachim Schmidt

**Affiliations:** 1 Department of Surgery, University Hospital Erlangen, Erlangen, Germany; 2 Regional Centre for Blood Transfusion and Clinical Immunology, Aalborg Hospital, Aalborg, Denmark; 3 Department of Anaesthesiology, University Hospital Jena, Jena, Germany; 4 Department of Medical Microbiology and Immunology, University of Aarhus, Aarhus, Denmark; 5 Professor, Department of Surgery, University Hospital Erlangen, Erlangen, Germany; 6 Department of Anaesthesiology, University Hospital Erlangen, Erlangen, Germany

Following the publication of the above article [1], we noticed that the Authors' contributions section was incorrect and should read as follows:

MS and JS conceived of the study, developed the study design and carried out the statistical analysis. JR was responsible for patient recruitment and clinical follow up, sample collection and DNA-isolation and also carried out the MBL-serum measurements. RS performed the MBL-genotyping. MM participated in the PCT analysis. ST participated in the study design and data analysis. WH participated in the study design and drafting of the manuscript. All authors read and approved the final manuscript.
